# Validation of a Multi-Channel Ambient Sensor to Measure Vital Signs in Patients Within the Ward and at Home

**DOI:** 10.3390/s25041149

**Published:** 2025-02-13

**Authors:** Seok Ming Lim, Peixuan Li, Sabine Braat, Ye Htet Aung, Susan Fu, Douglas F. Johnson, Wen Kwang Lim

**Affiliations:** 1 Department of Medicine, Faculty of Medicine, Dentistry and Health Sciences, The University of Melbourne, Melbourne, VIC 3053, Australia; 2 The Royal Melbourne Hospital, 300 Grattan Street, Melbourne, VIC 3050, Australia; 3 Centre for Epidemiology and Biostatistics, Melbourne School of Population and Global Health, The University of Melbourne, Melbourne, VIC 3053, Australia; 4 Methods and Implementation Support for Clinical Health (MISCH) Research Hub, Faculty of Medicine, Dentistry and Health Sciences, The University of Melbourne, Melbourne, VIC 3053, Australia

**Keywords:** ambient sensor, remote monitoring, patients

## Abstract

Hospitalised, unwell patients have vital signs such as heart rate (HR), oxygen saturation (SpO_2_) and temperature measured multiple times a day to detect clinical deterioration and monitor health trajectories. Advancements in contact-free (ambient) sensors (AS) to measure vital signs can help mitigate risks due to skin injury and infection transmission seen in conventional hospital equipment. This prospective, observational clinical study aims to validate vital sign measurements from a multi-channel AS compared to conventional equipment in three cohorts: patients in a hospital ward, patients at home within a Hospital-at-Home service, and healthy volunteers. Data analysis of 571 paired measurements from 29 participants indicates that heart rate measurements via AS were accurate, though they lacked precision, with the clinical agreement range between 6.38 and 6.49 beats per minute. Temperature and oxygen saturation measurements showed less agreement when compared with the reference standard. In conclusion, ambient sensors show promising utility in measuring vital signs, with this study amongst the first of its kind to utilise this in measuring vital signs in hospitalised patient cohorts in both ward and home environments.

## 1. Introduction

Vital signs such as respiratory rate, blood pressure, heart rate (HR), oxygen saturation (SpO_2_) and temperature are routinely measured four times a day amongst hospitalised patients to assess their physiological status and clinical trajectory [1]. Unwell patients are monitored more frequently as a means of maintaining patient safety with earlier detection of clinical deterioration [2,3]. Such intensive monitoring has traditionally only been able to be conducted within hospital wards. However, the increasing burden of multi-morbidity amid an aging populace globally has raised concerns around the sustainability and effectiveness of current models of hospital-based healthcare [3]. Changing work patterns and workforce shortages after the Coronavirus disease 2019 (COVID-19) pandemic have further exacerbated issues in the equitable delivery of healthcare [4].

As such, healthcare systems have had to explore novel methods to manage hospital bed pressures amid a mismatch in demand, capacity and resources. ‘Hospital-at-Home’ (HaH) services have been proposed as a way of managing these resource limitations, in addition to benefits such as reduced risk of infectious and iatrogenic complications, increased patient and carer satisfaction and decreased treatment costs [5,6,7]. HaH delivers “acute inpatient equivalent care, utilising highly skilled staff, hospital technologies, equipment, medical, and safety and quality standards, to deliver hospital-level care within a person’s place of residence of preferred (non-hospital) treatment location” [8].

Another recent development in healthcare relates to technological advancements in contact-free sensors (also called ambient sensors (AS)) to measure vital signs, by utilising infra-red sensors to measure body temperatures and red/green/blue (RGB) light cameras for SpO_2_ measurements [9,10,11]. These can mitigate risks and problems inherent in conventional contact-based measuring devices which are associated with infection transmission between patients or to staff and patients, and issues of skin irritation, pressure-related injuries and loss of data due to loss of contact between the patients’ skin and the device [3,12]. However, despite being shown to be feasible in an experimental or lab-based setting, there are minimal studies investigating the application of AS in clinical practice. Consequently, there is a need for clinical studies to validate the utility of AS for measuring vital signs amongst inpatients in hospital settings.

In this hospital-led study, we aim to compare vital sign measurements from a single multi-channel, remotely connected AS to conventional vital sign measuring devices. We hypothesised that HR, SpO_2_, and body temperature measured via the AS would agree with measurements obtained utilising conventional hospital equipment among hospitalised inpatients. In doing so, we aim to delineate the utility of AS for measuring vital signs in unwell patients within a hospital ward and for remote monitoring of vital signs amongst home-based patients in a HaH service.

## 2. Materials and Methods

### 2.1. Study Setting and Participants

This pragmatic prospective observational cross-sectional study was conducted in a tertiary metropolitan health service in Melbourne, Australia between 1 April 2023 and 1 April 2024. The study occurred within three settings: inpatients within the Acute Care of the Elderly (ACE) ward, home-based patients within the hospital’s HaH service, and healthy volunteers, as these are targeted populations most likely to benefit from the AS device. Elderly patients in the ACE ward are a frail, multi-morbid population over the age of 65 years who are vulnerable to iatrogenic complications of infection transmission and skin injuries from contact-based sensors. HaH patients are an acutely unwell population cared for by home-visiting hospital staff within an ‘early supported discharge’ model of care following admission to hospital [7]. These patients are located geographically remote from hospital personnel whereby the AS could assist in providing supplemental and on-demand vital sign measurements. Healthy volunteers are a well population with no known medical conditions. These volunteers serve as a comparison given the variability in clinical status of the two patient populations.

Inclusion criteria for patients include an estimated length of stay in the ward for a minimum of 5 days and sufficient manual dexterity and digital literacy for HaH articipants to use the AS and companion application to send vital sign measurements to the web-based platform. All study participants were 18 years of age or older and willing and able to provide informed consent for participation in the study.

Exclusion criteria included patients with infectious diseases or clinical conditions requiring isolation, and patients with other clinical concerns, such as delirium or behavioural disturbances, that would preclude inclusion in the study, deviate from usual clinical monitoring processes, or prevent the patient from completing the study. No exclusion criteria applied to the healthy volunteers.

### 2.2. Study Procedures

Clinicians within each study setting identified potentially eligible participants within 48 h of admission based on the study’s inclusion and exclusion criteria. Written consent was obtained following the provision of written and verbal information. The healthy volunteer cohort was recruited from hospital staff. Participants were advised that they could withdraw from the study at any time. The research team could also withdraw a participant if they acquired any exclusion criteria. Each participant received concurrent measurements from AS and RS for up to 5 days, or until leaving the study settings, whichever occurred first. Healthy volunteers were in the study settings for one day; hence, they received a single spot measurement from AS and RS simultaneously.

At the start of the study, the research team educated participants and nursing staff about utilising AS and was not involved in obtaining subsequent measurements from AS. Participants within the ACE ward had nursing staff use AS to measure vital signs; whereas HaH participants and healthy volunteers utilised AS themselves to measure their vital signs. In order to ensure measurements from AS and RS were as synchronous as possible, participants and nursing staff were educated to first set up conventional equipment for RS measurements before setting up the AS. They were then instructed to record measurements from both RS and AS simultaneously. A maximum time gap of 5 min between AS and RS measurements was considered acceptable for inclusion in the dataset, as the gap typically arose due to needing to adjust equipment placement. Measurements from RS were obtained from nursing staff in the ACE ward and HaH settings as part of routine clinical care. In contrast, study investigators were involved in obtaining these from healthy volunteers. Among hospitalised participants in the study, measurements from AS were conducted in parallel with routine hospital care but not used for clinical decision-making.

### 2.3. Devices: Multi-Channel Contact-Free Sensor (AS) and Conventional Hospital Equipment

**Figure 1:**
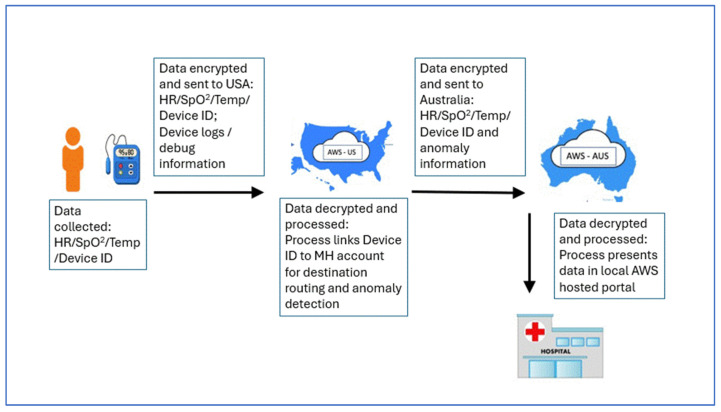
Data flow from ambient sensor to tablet application and web-based platform. [ID = Identity; USA = United States of America; AWS = Amazon Web Services; MH = Melbourne Hospital; Aus = Australia; HR = Heart Rate; SpO_2_ = oxygen saturation; Temp = Temperature].

The study utilised a prototype system (Norbert Health, New York, NY, USA) consisting of a portable AS, a patient-facing tablet application, secure cloud-based data-hosting and processing, and web-based dashboard, with data flowing from the AS to the web-based dashboard as depicted in Figure 1. The AS utilises three built-in sensors linked to an on-board computer and clock that integrates the signals with algorithms to calculate measurements of HR, SpO_2_ and temperature. The three sensors consist of a Red/Green/Blue video camera, an mmWave radar sensing unit and a thermal infrared camera. The video camera uses remote photoplethysmography (rPPG), which is a contactless technique that converts light signals from colour changes on the face and skin surface into electrical signals known as rPPG signals. The radar sensing unit transmits and receives mmWave signals reflected off the skin surface, taking into account micro-motions due to participants’ pulse and respiration. The thermal camera detects infrared radiation from the skin surface and converts this to a thermogram. Neural networks within the onboard computer analyse these signals to calculate HR, SpO_2_ and peripheral body temperature.

To utilise the AS, the participant’s face and hand are placed approximately 1.2 m away from the camera for a 30–45 s period. The three sensors measure data from skin surface movement, colour, and infrared energy, which are then subsequently transformed into HR, SpO_2_ and temperature measurements. Measurements are then transmitted from the AS via Bluetooth to the application within the paired tablet device, with subsequent transference in real-time via cellular network to a secure cloud server for hosting and analysis. Measurements are displayed on a web-based dashboard accessible to study investigators for review.

Conventional hospital equipment utilised as reference standard equipment (RS) includes a pulse oximeter to measure SpO_2_ and HR (Phillips SureSigns VS4 monitor, Phillips Electronic Australia Limited, Melbourne, Australia) and tympanic thermometer (Welch Allyn Braun Thermoscan Pro6000, Covidien Genius 2 Thermometer, Welch Allyn Australia Pty Limited, Melbourne, Australia). Both devices have prior validation via Therapeutic Goods Administration (TGA) for medical devices and are operated manually. The pulse oximeter uses a clip-like device placed on the patient’s finger for approximately a minute, with SpO_2_ and HR measurements obtained by calculating light absorption across the patient’s fingernail and finger. The thermometer uses infrared to measure temperature when placed within the patient’s external auditory canal. These devices are routinely tested and validated annually by the hospital’s bioengineering team.

#### Medical Device Regulation

The ambient sensor used in the study was not approved as a medical device. The prototype device had no Conformite Europeenne (CE) mark or FDA clearance and was designated for clinical investigation use only.

### 2.4. Outcomes

The primary outcome measures were individual vital sign measurements of HR, SpO_2_ and temperature of AS compared with the RS. HR was measured as beats per minute (bpm), SpO_2_ as percentage (%), and temperature in Celsius (°C). For each vital sign, clinically relevant differences between AS and RS were considered as follows: 10+/− bpm (HR); 2+/−% (SpO_2_); 5+/− °C (temperature). Data was analysed by time of day (daylight or evening) to determine the contribution of ambient lighting to the accuracy of the measurement.

### 2.5. Statistical Analyses

#### 2.5.1. Data Collection

Study data were collected and managed using REDCap electronic data capture tools hosted by the hospital’s Health Intelligence Unit [13]. Only study investigators had access to patient-identifiable information, with all study data subsequently anonymised before analysis by assigning a study ID to each participant. Data from the ambient sensor on the web-based dashboard were manually transcribed into REDCap by study investigators after the device ID was linked to the study ID of each participant.

#### 2.5.2. Statistical Methods

We planned to recruit a convenience sample of 10 participants from each setting—totalling 30 participants, with the aim of obtaining a minimum of 200 paired samples of HR, SpO_2_ and temperature measurements. This sample size was informed by the United States of America Food and Drug Administration (U.S. FDA) guidelines requiring at least 200 measurements from at least 10 subjects to validate the pulse oximeters [14]. Due to organisational challenges amid impacts post COVID-19, we were unable to recruit 10 patients from the HaH program; hence, the study ended after recruiting 29 participants, comprising 11 from the ACE ward, 8 from the HaH service, and 10 healthy volunteers.

Patient demographics were summarised using descriptive statistics, consisting of counts and frequencies for categorical data, and mean and standard deviation (SD, and/or median and interquartile range (25th to 75th percentile) or range (minimum to maximum) for continuous data.

The Bland and Altman method was used to analyse data pairs of HR, SpO_2_ and temperature measurements obtained from AS and RS; in obtaining the mean difference between measurements, upper and lower limits of agreement (LoA), and corresponding two-sided 95% confidence intervals (CI) [15,16]. ACE ward patients and HaH patients had multiple observations per individual, therefore we assumed that the actual value varied in each participant (i.e., measurements were made under different conditions over the observational period) [17]. Standard deviation was calculated using the within-subject and between-subject variances [17]. The MOVER method was used to estimate the 95% CIs of LoA for repeated measurements [17]. Healthy volunteers had a single measurement, thus allowing the application of the original Bland and Altman methodology [16]. If underlying assumptions were violated, Cohen’s Kappa coefficient was used to quantify the level of agreement between AS and RS [18]. In addition, we obtained the root mean squared error (also referred to as ARMS) for all vital signs, which is related to bias and precision and corresponds to the clinical agreement range between devices [19]. All analyses were performed using STATA/SE version 17.0 (StataCorp, College Station, TX, USA).

### 2.6. Ethics

Ethics approval was obtained from the Melbourne Health Human Research Ethics Committee (reference number HREC/89542/MH-2022). The findings from this study are reported according to the Guidelines for Reporting Reliability and Agreement Studies [20].

## 3. Results

### 3.1. Participant Demographics

Participant demographics and characteristics across the three cohorts from the ACE ward, HaH patients and healthy volunteers are depicted in Table 1. Most participants had Fitzpatrick skin types 1 or 2, corresponding with a lighter degree of pigmentation [21]. Participants ranged in age from 25 to 100 years, and testing occurred amongst patients with a variety of acute medical and surgical conditions, such as sepsis, exacerbation of heart and lung disease, falls and fractures, and post-surgical complications. Hospitalised participants had a median age of 82 years, with Clinical Frailty Scale (CFS) ranging from 2 to 5; consistent with a vulnerable cohort experiencing symptoms that limit daily activities [22]. Home-based participants were mostly middle-aged (median age 56 years) and more independent, with a median CFS score of 2.5. Cardiovascular disease was the most common comorbidity in both ACE and HaH participant groups, with the hospitalised cohort being more comorbid in comparison to the home-based cohort.

### 3.2. Validation of Vital Sign Measurements

There were 571 paired data points of vital sign measurements comparing the AS against the RS. Vital signs, including HR, SpO_2_ and temperature, were compared, and measurement data from AS and RS were validated across the three cohorts: the ACE ward, HaH patients, and healthy volunteers (Table 2). Participants from the ACE ward had the highest number of paired measurements in comparison to HaH and volunteer cohorts; this corresponds to the frequency with which hospitalised patients would have undergone routine vital sign measurements on the ward compared to being in the home environment.

#### 3.2.1. Heart Rate

**Figure 2:**
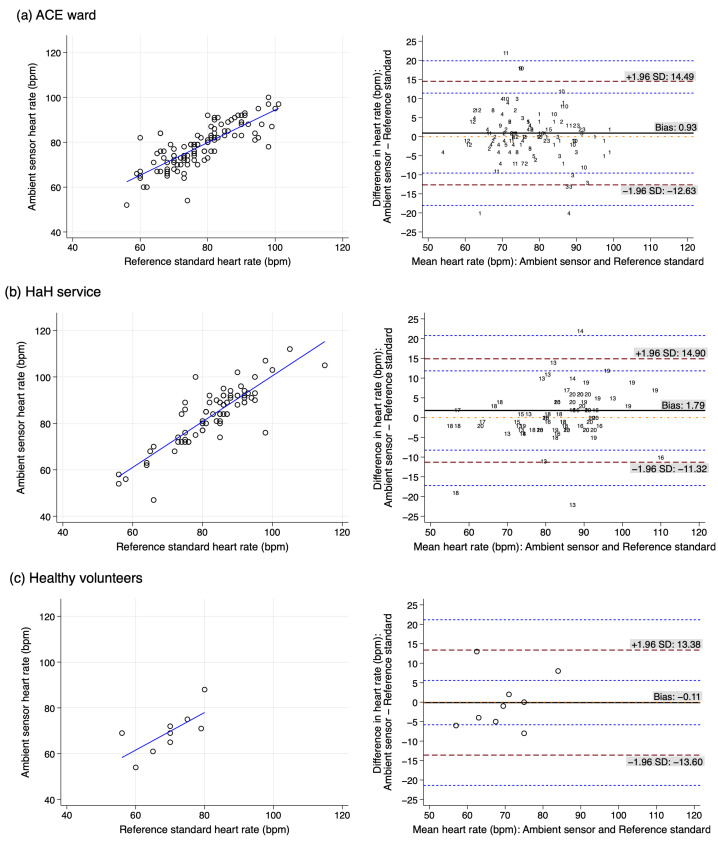
Scatter plots and Bland–Altman analyses of heart rate measurements in ambient sensor with the reference standard amongst participants from (**a**) ACE ward, (**b**) HaH service and (**c**) healthy volunteers. Numbers are used in (**a**,**b**) to represent multiple observations for individual participants. (The dashed red lines are the upper limit of agreement and lower limit of agreement (from top to bottom); the solid black line is bias; the dashed blue lines are the 95% confidence intervals around the upper limit of agreement and lower limit of agreement; the dotted orange line represents perfect agreement. Circles represents individual observations.).

Heart rate data from 193 paired measurements from 28 participants were analysed as shown in Figure 2. Amongst participants in the ACE ward, HAH service and healthy volunteers Bland–Altman plots suggest a constant bias, with calculations indicating the bias was +0.93 bpm (95% confidence interval (CI) −0.47, 2.33), +1.79 bpm (95%CI −0.19, 3.77) and −0.11 bpm (95%CI −5.40, 5.18) respectively. As indicated by ARMS, the clinical agreement range was 6.38 bpm in the ACE ward cohort, 6.46 bpm in the HaH service cohort, and 6.49 bpm for healthy volunteers. Amongst participants from the ACE ward and HaH service, comparison of daytime measurement results to evening data showed a positive bias of +1.26 bpm and ARMS of 6.57 bpm for the ACE ward, compared to +0.87 bpm and 4.19 bpm for the HaH service (Figure S1 within Supplementary Materials).

#### 3.2.2. Oxygen Saturation

**Figure 3:**
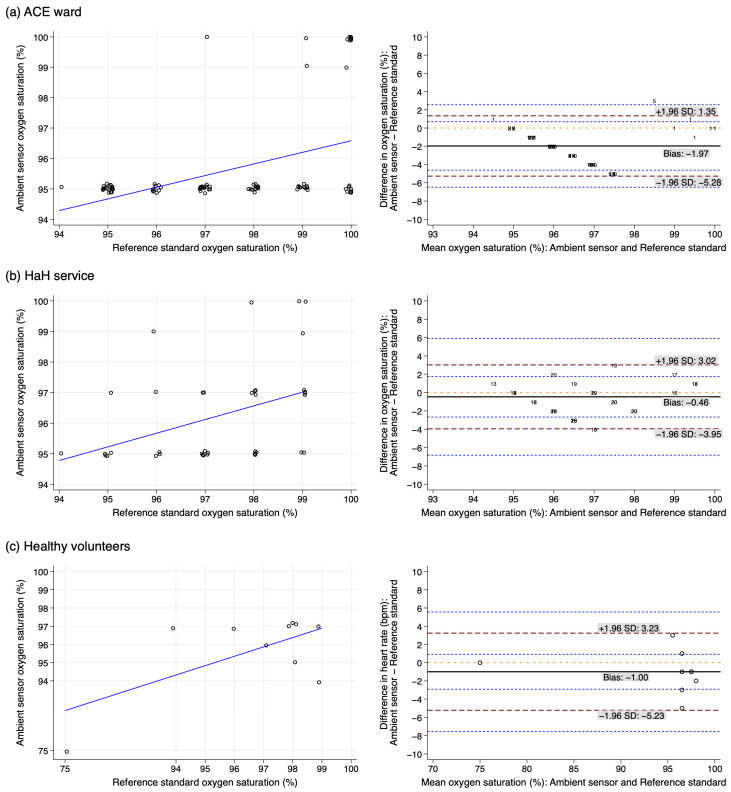
Scatterplots and Bland–Altman analyses of oxygen saturation measurements in the ambient sensor with the reference standard amongst participants from (**a**) ACE ward, (**b**) HaH service and (**c**) healthy volunteers. Numbers are used in (**a**,**b**) to represent multiple observations for individual participants. Scatterplot with jitter applied to display repeated values. (The dashed red lines are the upper limit of agreement and lower limit of agreement (from top to bottom); the solid black line is bias; the dashed blue lines are the 95% confidence intervals around the upper limit of agreement and lower limit of agreement; the dotted orange line represents perfect agreement. Circles represents individual observations.).

Paired measurements of 139 SpO_2_ values from 27 participants were analysed as shown in Figure 3. For SpO_2_ measurements, the range of measurements of the AS and RS were seemingly different, with a non-constant bias, thus violating the assumptions of the Bland–Altman analysis (Figure 3). A general observation is that the majority of ward measurements using the AS indicated a 95% SpO_2_ whilst the RS ranged from 94–100% (Figure 3 and Table S1 within Supplementary Materials). More variability in the measurements was observed in the home-based setting, with Cohen’s Kappa coefficient of 0.03 amongst 8 home-based patients compared with Cohen’s Kappa coefficient of 0.12 amongst 9 ward patients. Similarly, the 10 healthy volunteers had a Cohen’s Kappa coefficient of 0.01.

#### 3.2.3. Temperature

**Figure 4:**
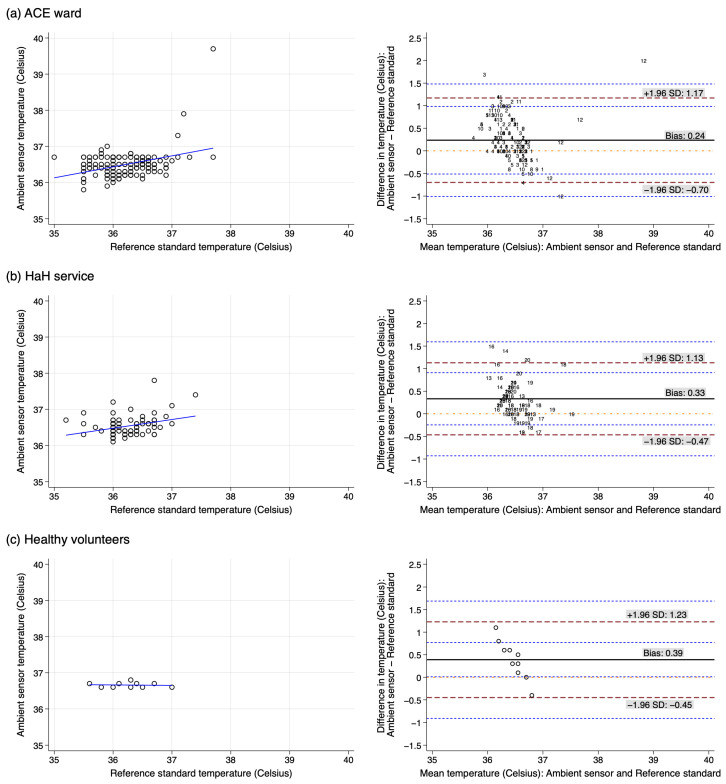
Scatterplots and Bland–Altman analyses of temperature measurements in ambient sensor with the reference standard amongst participants from (**a**) ACE ward, (**b**) HaH service and (**c**) healthy volunteers. Numbers are used in (**a**,**b**) to represent multiple observations for individual participants. (The dashed red lines are the upper limit of agreement and lower limit of agreement (from top to bottom); the solid black line is bias; the dashed blue lines are the 95% confidence intervals around the upper limit of agreement and lower limit of agreement; the dotted orange line represents perfect agreement. Circles represents individual observations.).

The temperature of 239 paired measurements from 29 participants was analysed as shown in Figure 4. The Bland–Altman plot suggested a negative trend of differences between AS and RS with increasing average temperature indicating a non-constant bias, thus violating the underlying assumptions (Figure 4). The AS values ranged from 35.8 to 39.7 degrees Celsius while the RS ranged from 35 to 37.7 degrees Celsius (Figure 4). The ARMS for the ward participants, HaH participants, and healthy volunteers revealed that the ARMS was 0.54 degrees Celsius, 0.48 degrees Celsius and 0.36 degrees Celsius, respectively. Amongst participants from the ACE ward and HaH service, measurements taken in daylight revealed ARMS of 0.53 degrees Celsius, compared to evening measurements with an ARMS of 0.45 degrees Celsius.

## 4. Discussion

In this study, we compared measurements from an AS to conventional ward-based equipment across two clinical settings (elderly ward and home-based HaH patients) and amongst healthy volunteers. Results indicate that HR measurements by AS were accurate but lacked precision; with bias ranging from −0.11 bpm to +1.79 bpm in the three cohorts. Though this was a small study with limitations, as noted below, our study appears to be the first instance in which a multi-channel AS has been validated against conventional hospital equipment for measurement of vital signs amongst unwell patients. It involved participants across a spectrum of ages from 25 to 100 years and with a variety of clinical illnesses.

Temperature and oxygen saturation measurements via AS showed less agreement than those of RS. There is an increasing understanding that age significantly impacts rPPG performance; hence it is possible our measurements were impacted by the high proportion of elderly patients within our study [23]. Increasing age is associated with increased variability in skin tone and folds; additionally, pigmentation changes, moles, and wrinkles can affect the facial recognition technology utilised within camera sensors [24]. Datasets commonly used in facial recognition technologies remain limited to narrow demographic compositions, with the majority consisting of healthy young adult (aged 18–30 years) Caucasian subjects [24,25,26]. Datasets within conventional pulse oximetry systems have had similar concerns raised, due to increasing awareness of the potential impact of race, age and clinical conditions such as atherosclerosis on data interpretation [27,28,29]. Our study’s findings complement the growing awareness around the importance of increasing the diversity of datasets in order to minimise algorithmic biases when developing prototypical new technologies. Besides potentially improving accuracy of temperature and SpO_2_ measurements, the precision of HR measurements would likely be increased. Importantly, health technologies need to consider the effects of advanced age and end-organ dysfunction, as these are representative of patients within our health system.

Our study corroborates and adds to the available literature regarding the complexities of evaluating the clinical accuracy of infrared thermography for measuring body temperature [30,31]. Temperatures at peripheral body sites can differ and fluctuate depending on ambient temperature, activity levels, metabolic rate and hormonal changes. These complexities in human thermoregulation potentially impacted our study, which was conducted across multiple locations and time of day [32]. Although the hospital’s tympanic membrane thermometer is a well-established and validated clinical reference equipment used for daily patient care, it is also recognised that tympanic membrane thermometry can be negatively impacted by dirt/cerumen and inaccurate placement of the thermometer [31,33]. Future studies should attempt to control for these methodological and physiological confounders that may affect the accuracy of non-contact infrared thermometry.

The development of an AS that accurately monitors HR has significant potential to improve care in multiple settings. Various heart rate indices such as heart rhythm, resting heart rate and heart rate variability, are surrogate markers for physiological and cardiac health. These indices have been shown to be useful in predicting arrhythmic events, sudden cardiac death, and heart failure management [34,35]. Unlike wearable devices or conventional vital sign-measuring equipment, the ease and non-intrusive nature of ambient monitoring facilitate continuous data capture while the patient conducts their daily activities. For the HaH cohort, continuous remote monitoring via placement of an AS within patients’ home environments can improve patient safety and prevent serious issues by earlier detection of, and rapid institution of treatment for, clinical deterioration in the community.

We acknowledge several limitations in our study arising from its clinical setting and the preliminary nature of our results. Recruitment was impacted by COVID-19 and was opportunistic and pragmatic, whereby patients who were unable to consent due to either being clinically too unstable or lacking in digital literacy did not participate in the study. Due to organisational challenges, there were fewer paired observations collected than planned. Though the slightly reduced sample size is unlikely to significantly affect the statistical significance of the results, there might be wider confidence intervals around the observed bias and limits of agreement. This needs to be addressed via a larger follow-up study. A second and potentially more important limitation is that our participants’ vital signs were largely within physiologically normal ranges even though they were acutely unwell and undergoing treatment as inpatients. For example, the distribution of reference temperatures was uneven, with a limited number of participants having elevated body temperatures. An optimal dataset would provide a more uniform distribution of HR, SpO_2_ and temperatures across both abnormally low and high, and normal physiological ranges. It was not possible within our study to recruit participants with vastly abnormal vital signs due to consequent clinical instability, and we elected to focus our investigations amongst elderly and HaH cohorts due to the paucity of research in these settings [36,37]. Lastly, a strength and limitation of our study lies in the investigation of outcomes within the HaH cohorts, where it was not possible to control for ambient lighting, temperature and background movement amongst the participants in their own homes. This adds complexity, as the algorithms interpreting data from the AS could be affected by inadequate selection or detection of regions of interest or insufficient noise reduction strategies when interpreting rPPG signals. Future research should focus on testing novel sensors across different clinical scenarios and patient cohorts in order to improve sensitivity in detecting clinical deterioration.

## 5. Conclusions

In conclusion, multi-channel ambient sensors show promising utility in measuring vital signs amongst hospitalised patient cohorts in both ward and home environments, with heart rate measurements being more accurate than other domains measured in our study. Ambient sensing can reduce the burden on patients and healthcare providers by non-intrusively monitoring and measuring vital signs in place of conventional ward equipment. Our preliminary results underline the importance of testing novel sensor technologies in diverse datasets and across different patient cohorts. Future studies could incorporate multi-centre trials assessing different vital sign domains within a larger cohort and across different clinical and disease settings.

## Figures and Tables

**Table 1:** Demographics and summary of vital sign measurements of participants by study setting.

		Ward (n = 11)	Home-Based (n = 8)	Volunteers (n = 10)
Age (years), median (range)		82 (76–100)	56 (25–79)	35 (29–62)
Gender—Male (n, %)		5 (46)	5 (63)	6 (60)
Skin type—Fitzpatrick scale, median (range)		1 (1–3)	1 (1–2)	1 (1–3)
Clinical Frailty Scale, median (range)		4 (2–5)	2.5 (1–4)	1 (1–3)
Relevant medical history (n *, %)				
	No relevant history	0	2 (25)	10 (100)
	Cardiovascular disease	10 (91)	5 (63)	0
	Chronic respiratory disease	6 (55)	1 (13)	0
	Atrial fibrillation	3 (27)	0	0
	Diabetes mellitus	3 (27)	3 (38)	0
	Malignancy	1 (9)	0	0
	Connective tissue disease	1 (9)	4 (50)	0
Reason for hospitalisation (n, %)				
	Acute cardiac illness	3 (27)	1 (13)	N/A
	Fall	5 (46)	0	N/A
	Sepsis	0	5 (63)	N/A
	Surgery	1 (9)	4 (50)	N/A
	Malignancy-related	1 (9)	0	N/A
	Other	1 (9)	0	N/A

* Participants might have more than one medical condition, with the total sum of relevant medical history not corresponding to the number of participants. N/A = Not Applicable.

**Table 2:** Summary of paired vital sign measurements of participants by study setting.

	Ward		Home-Based		Volunteers	
	AS	RS	AS	RS	AS	RS
Heart Rate (bpm)	(n = 11)		(n = 8)		(n = 9)	
Number of observations	111	111	73	73	9	9
Mean (SD)	78.2 (9.5)	77.7 (10.3)	83.6 (12.5)	82.9 (10.9)	69.3 (9.4)	69.4 (8.1)
Median (IQR)	78 (72–84)	76 (70–84)	85 (76–92)	85 (76–90)	69 (65–72)	70 (65–75)
Range	52–100	56–101	47–112	56–115	54–88	56–80
SpO_2_ (%)	(n = 9)		(n = 8)		(n = 10)	
Number of observations	90	90	39	39	10	10
Mean (SD)	95.6 (1.6)	97.4 (1.8)	96.3 (1.6)	97.3 (1.5)	94.2 (6.8)	95.2 (7.3)
Median (IQR)	95 (95–95)	97 (96–99)	95 (95–97)	98 (96–99)	97 (95–97)	98 (96–98)
Range	95–100	94–100	95–100	94–99	75–97	75–99
Temperature (°C)	(n = 11)		(n = 8)		(n = 10)	
Number of observations	146	146	83	83	10	10
Mean (SD)	36.5 (0.4)	36.2 (0.5)	36.5 (0.3)	36.2 (0.4)	36.7 (0.1)	36.3 (0.4)
Median (IQR)	36.5 (36.3–36.7)	36.2 (35.9–36.5)	36.5 (36.3–36.6)	36.1 (36.0–36.5)	36.7 (36.6–36.7)	36.3 (36.0–36.5)
Range	35.8–39.7	35–37.7	36.1–37.8	35.2–37.4	36.6–36.8	35.6–37

AS = Ambient Sensor; RS = Reference Standard; SD = Standard Deviation; IQR = Interquartile range.

## Data Availability

The data presented in this study are available on request from the corresponding author, subject to restrictions based on Australian legislation governing privacy and health data.

## Supplementary Materials

The following supporting information can be downloaded at: https://www.mdpi.com/article/10.3390/s25041149/s1, Figure S1: Scatter plots and Bland–Altman analyses of heart rate measurements in ambient sensor with the reference standard across ACE ward and HaH service participants measured during (a) daylight and (b) evening; Table S1: Oxygen saturation measurements in ambient sensor and the reference standard by study setting.
