# Supplementary material for: Validation of a Multi-Channel Ambient Sensor to Measure Vital Signs in Patients Within the Ward and at Home
Source: Sensors (Basel). 2025 Feb 13;25(4):1149. doi: 10.3390/s25041149 (PMC11859041; doi:10.3390/s25041149)
Supplement: Supplementary file 1 [file sensors-25-01149-s001.zip › sensors-3399117-supplementary.pdf]

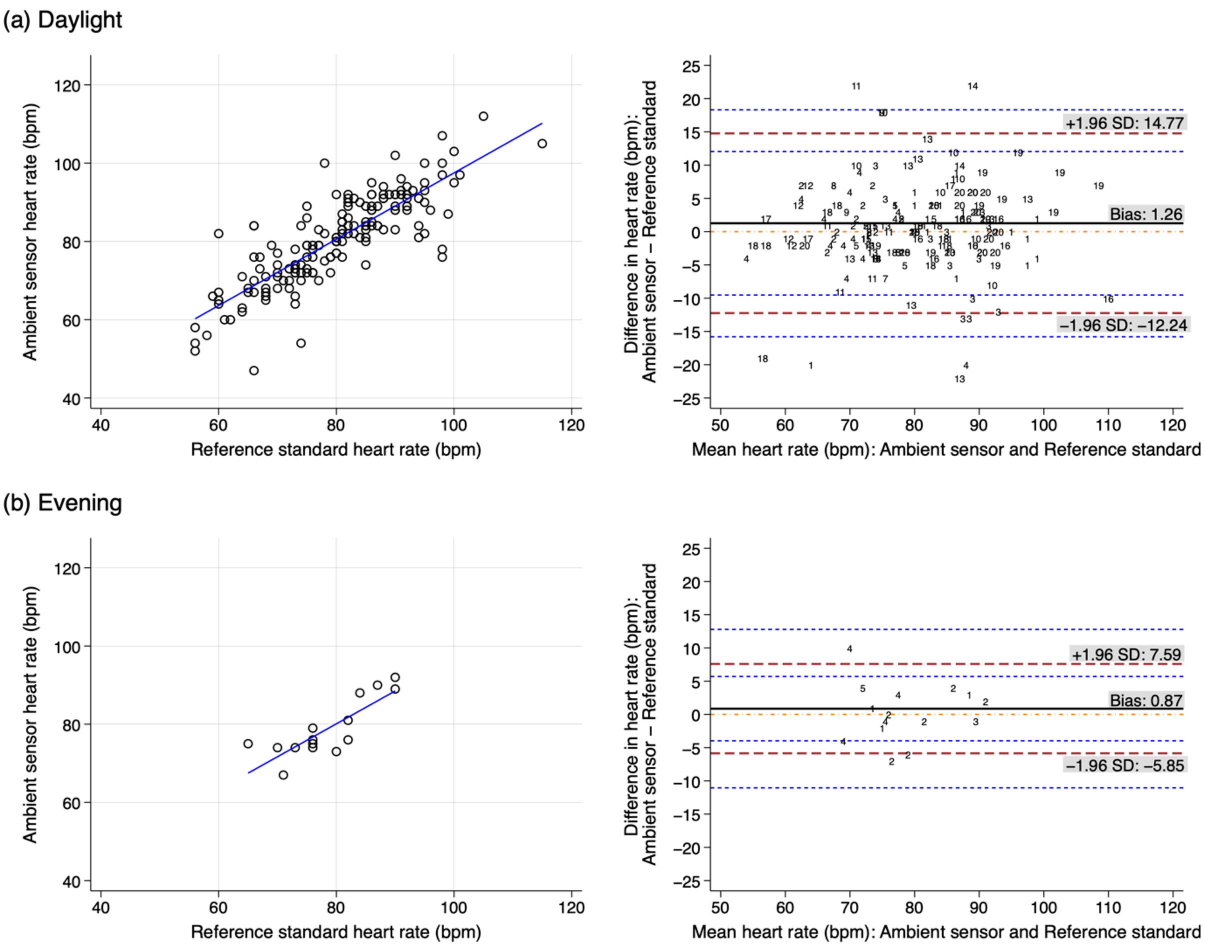

SD = Standard Deviation; bpm = beats per minute.

Figure S1. Scatterplots and Bland-Altman analyses of heart rate measurements in ambient sensor with the reference standard across ACE ward and HaH service participants measured during (a) daylight and (b) evening. Numbers are used in (a,b) to represent multiple observations for individual participants. *(The dashed red lines are the upper limit of agreement and lower limit of agreement (from top to bottom); the solid black line is bias; the dashed blue lines are the 95% confidence intervals around the upper limit of agreement and lower limit of agreement; the dotted orange line represents perfect agreement. Circles represents individual observations.)*

Table S1. Table of oxygen saturation measurements in ambient sensor with the reference standard by study setting.

**Ward patients**							
**Ambient sensor oxygen saturation (%)**	**Reference standard oxygen saturation (%)**						
	**94**	**95**	**96**	**97**	**98**	**99**	**100**
**94**	0	0	0	0	0	0	0
**95**	1	20	10	15	13	12	8
**96**	0	0	0	0	0	0	0
**97**	0	0	0	0	0	0	0
**98**	0	0	0	0	0	0	0
**99**	0	0	0	0	0	1	1
**100**	0	0	0	1	0	1	7

**HaH service**							
**Ambient sensor oxygen saturation (%)**	**Reference standard oxygen saturation (%)**						
	**94**	**95**	**96**	**97**	**98**	**99**	**100**
**94**	0	0	0	0	0	0	0
**95**	1	4	3	6	5	2	0
**96**	0	0	0	0	0	0	0
**97**	0	1	1	2	4	5	0
**98**	0	0	0	0	0	0	0
**99**	0	0	1	0	0	1	0
**100**	0	0	0	0	1	2	0

**Healthy volunteers**						
**Ambient sensor oxygen saturation (%)**	**Reference standard oxygen saturation (%)**					
	**75**	**94**	**96**	**97**	**98**	**99**
**75**	1	0	0	0	0	0
**94**	0	0	0	0	0	1
**95**	0	0	0	0	1	0
**96**	0	0	0	1	0	0
**97**	0	1	1	0	3	1
**98**	0	0	0	0	0	0
**99**	0	0	0	0	0	0
